# Common Clonal Origin of Core‐Binding‐Factor AML in Both Donor and Recipient Following Kidney Transplantation

**DOI:** 10.1155/crh/5549699

**Published:** 2026-06-26

**Authors:** F. E. M. in ’t Hout, M. Roerink, R. J. Maas, K. M. Hebeda, B. A. van der Reijden, W. J. F. M. van der Velden, S. M. C. Langemeijer

**Affiliations:** ^1^ Department of Hematology, Radboud University Medical Center, Nijmegen, the Netherlands, radboudumc.nl; ^2^ Department of Nephrology, Radboud University Medical Center, Nijmegen, the Netherlands, radboudumc.nl; ^3^ Department of Pathology, Radboud University Medical Center, Nijmegen, the Netherlands, radboudumc.nl; ^4^ Department of Laboratory Medicine, Laboratory of Hematology, Radboud University Medical Center, Nijmegen, the Netherlands, radboudumc.nl

## Abstract

Kidney transplant recipients are at higher risk of developing malignancies compared with the general population, which are mostly recipient in origin^1^. We describe a patient who developed a donor‐derived acute myeloid leukemia (AML) five years after kidney transplantation. His donor developed AML four years earlier, providing proof of transmission of leukemia‐initiating cells in solid organ transplant (SOT).

## 1. Case Description

**FIGURE 1:**
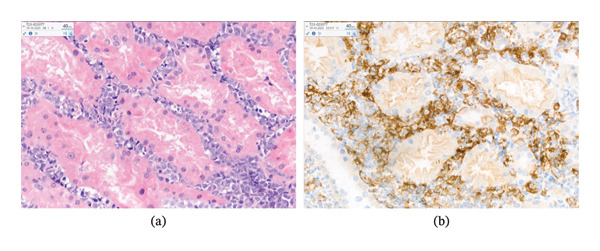
Biopsy of the donor kidney at disease onset 5 years after transplantation. (a) HE staining, 40x magnification. The interstitial space between the tubuli is filled with blasts. (b) CD117 immunohistochemistry identifying the blasts (brown).

A 55‐year old man received a non‐HLA‐matched living donor kidney transplantation for progressive renal failure due to autosomal dominant polycystic kidney disease. The patient and donor were not related, and there was no case of hematologic malignancy in their families. Five years after transplantation, under immunosuppressive medication consisting of tacrolimus (4 mg twice daily) and prednisone (10 mg daily), his kidney function decreased with a 1.5‐fold increase of the creatinine level. A biopsy of the allografted kidney showed a diffuse infiltration with myeloblasts, consistent with myeloid sarcoma (Figure 1). Blood counts were normal, but myeloblasts were seen on peripheral blood smears. In a bone marrow aspirate, 10% myelomonocytic blasts (French–American–British morphology M4) were detected. Remarkably, the cytogenetic analysis of affected cells showed a female karyotype with trisomy 8 and translocation t(16; 16)(p13.11; q22.1)/CBFB::MYH11. The acute myeloid leukemia (AML) was classified as AML with defining genetic abnormalities according to world health organization (WHO) [1], and AML with recurrent genetic abnormality according to the International Consensus Classification of AML (ICC) [2]. The presence of the female karyotype showed that the AML was of donor origin. No mutations were found in the AML panel (*ASXL1*, *CEBPA*, and *FLT3* (ITD or TKD), *KIT*, *NPM1*, *NRAS, RUNX1*, *TP53*, *IDH1*, or *IDH2*) using error‐corrected next generation sequencing (NGS) [3]. Sequencing showed DNA polymorphisms with abnormal variant allele frequencies indicative for a mixed chimeric pattern in line with the female karyotype in the AML cells. Based on the genetic and molecular characteristics, the patient is defined as good‐risk AML. The patient started intensive chemotherapy (cytarabine and daunorubicin), and tactrolimus was discontinued. The first course of treatment was complicated by graft failure, and the patient had to resume dialysis. Despite the complicated treatment, he had a MRD‐negative complete response. Currently, the patient is two years after completing this treatment and still in complete remission. He is on the waiting list for a new renal transplant.

**FIGURE 2:**
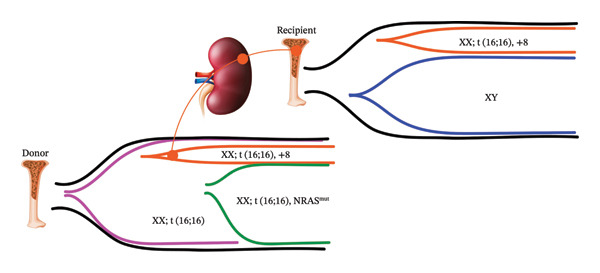
Clonal composition of donor and recipient.

At the time of kidney donation, the donor was a healthy 41‐year‐old woman with a mild anemia (Hb: 11.1 g/dL), presumably due to folic acid deficiency for which she received folic acid orally. Seven months after the kidney donation, she presented with fatigue, mucosal bleedings, and hematomas. Laboratory analysis of the peripheral blood revealed hyperleukocytosis (109 ∗ 10^9^/L), anemia (Hb 7.7 g/dL), and thrombocytopenia (20∗10^9^/L). Lactate dehydrogenase (LDH) was elevated (548 U/L). She was admitted to the intensive care unit with respiratory failure due to pulmonary leukostasis. Bone marrow aspirate analysis revealed myeloid blasts with a phenotype CD45+‐/SS‐CD34+CD117+HLADR + CD38+CD13+CD11b‐CD133+‐CD64‐, matching a myelomonoblastic phenotype. Cytogenetic findings consisted of trisomy 8 (10%–15% of the cells) and translocation t(16; 16)(p13.11; q22.1)/CBFB::MYH11 (97% of the cells) identical to the genetic abnormalities found in the recipient AML. Additionally, NGS revealed a *NRAS* mutation (VAF 38%), which was not detected in the recipient AML (Figure 2). There were no other mutations found in the AML panel. A comparison of polymorphisms showed that all donor polymorphisms were clearly detected in the bone marrow of the kidney recipient.

At diagnosis, the kidney donor was classified as intermediate‐risk AML because the t(16; 16)(p13.11; q22.1)/CBFB::MYH11 was only found retrospectively. She was treated with one course of intensive chemotherapy (cytarabine and idarubicin), which was, due to toxicity, followed by 2 cycles of 5‐day decitabine 20 mg/m^2^. She achieved a measurable residual disease‐negative complete remission as shown by flow cytometry. She underwent a reduced‐intensity allogeneic hematopoietic cell transplantation (HCT) with a matched unrelated donor. To date, four years after HCT, she is alive and in remission with complete donor chimerism (sensitivity of 0.1%) at the last follow‐up.

## 2. Discussion

We describe a rare occurrence of CBF‐AML in both donor and recipient developing from the same ancestral clone, indicating that the malignant cells had been present in the kidney during the transplantation. The leukemic cells in the recipient, however, took four more years to develop into AML compared with the leukemic cells in the donor, which might be due to cells residing in an extramedullary niche within the allograft, the host‐versus‐leukemia immune reaction of the recipient vs. donor and the absence of the *NRAS* mutation (which is associated with a highly proliferative AML) in the patient. Marchionni et al. described AML development in 3 recipients post solid organ transplantation from one female donor [4]. Here, mutations in *NPM1*, *RUNX1*, *TET2*, and *DNMT3A* were present in the donor and shared in all recipients. All recipients also had specific additional mutations [4].

Our patient and recipient both had t(16; 16)(p13.11; q22.1)/CBFB::MYH11 and a trisomy 8 and myelomonocytic morphology. Myelomonocytic AML is recognized as a subtype of AML, which is correlated with extramedullary disease [5]. While the donor harbored a *NRAS* mutation in her AML, this was not detected in the recipient. The inv(16) may be an early event in AML development. Possibly, the donor developed the additional *NRAS* mutation after kidney donation. In the recipient, this *NRAS* mutation was not detected in the dominant clone; however, due to the low blast count in the bone marrow, a subclone with an *NRAS* mutation (VAF < 1%) could not be excluded.

Donor‐derived myeloid neoplasms after solid organ transplant (SOT) are extremely rare [4, 6]. They originate from either clonal hematopoietic stem or progenitor cells seeding the transplanted organ and subsequently evolve into AML by acquiring additional genetic abnormalities in an often proinflammatory environment. Alternatively, overt leukemic cells are transplanted with the solid organ. In both scenarios, the presence of a HLA mismatch might prevent early clonal expansion due to a host‐versus‐leukemia immune response, but eventually, chronic immunosuppressive therapy precludes definite clonal eradication.

Currently, it is not known if or how many hematopoietic progenitor cells are present in an organ transplant. Extramedullary hematopoiesis can be found in spleen, liver, and lymph nodes; however, it has also been described in the heart, fat tissue, adrenal glands, and kidney but mainly in association with (benign) hematopoietic disorders or cancer [7]. Interestingly, multilineage hematopoiesis resulting in full donor chimerism was described in a pediatric kidney transplant recipient with syndromic‐combined immune deficiency [8]. This supports the possibility of kidneys containing sufficient hematopoietic stem cells to support long‐term and multilineage hematopoiesis.

In conclusion, we describe the unusual transplantation of leukemia‐initiating cells through SOT with a different clonal evolution between donor and recipient AML. Because of the extremely rarity of the event, we deem screening for leukemic cells in SOTs unfeasible. However, in case of unexplained decrease in organ function or aberrations in peripheral blood counts in the recipients, further investigation is warranted.

## Author Contributions

F. E. M. in ’t Hout and S. M. C. Langemeijer wrote the manuscript. M. Roerink, R. J. Maas, and W. J. F. M. van der Velden were involved in the clinical work. K. M. Hebeda and B. A. van der Reijden were involved in the diagnostics. All authors were involved in the interpretation of the case and gave critical feedback and helped shape manuscript.

## Funding

No funding was received for this manuscript.

## Consent

Patients involved consented with publication by signing a HEMBB01 consent.

## Conflicts of Interest

The authors declare no conflicts of interest.

## Data Availability

Data sharing is not applicable to this article as no datasets were generated or analyzed during the current study.
